# Structural connectivity in ventral language pathways characterizes non-verbal autism

**DOI:** 10.1007/s00429-022-02474-1

**Published:** 2022-03-14

**Authors:** Guillem Olivé, Dominika Slušná, Lucía Vaquero, Jordi Muchart-López, Antoni Rodríguez-Fornells, Wolfram Hinzen

**Affiliations:** 1 Department of Cognition, Development and Educational Psychology, Campus Bellvitge, University of Barcelona, L’Hospitalet de Llobregat, 08097 Barcelona, Spain; 2 Department of Translation and Language Sciences, Campus Poblenou, Pompeu Fabra University, 08018 Barcelona, Spain; 3 Legal Medicine, Psychiatry, and Pathology Department, Faculty of Medicine, Complutense University of Madrid, 28040 Madrid, Spain; 4 Department of Neuroradiology, Hospital Sant Joan de Déu, Barcelona, Spain; 5 Cognition and Brain Plasticity Group, Bellvitge Biomedical Research Institute, L’Hospitalet de Llobregat, 08097 Barcelona, Spain; 6 Institució Catalana de Recerca i Estudis Avançats, ICREA, 08010 Barcelona, Spain

**Keywords:** MRI, DWI, Tractography, Autism, Non-verbal, Language

## Abstract

Language capacities in autism spectrum disorders (ASD) range from normal scores on standardized language tests to absence of functional language in a substantial minority of 30% of individuals with ASD. Due to practical difficulties of scanning at this severe end of the spectrum, insights from MRI are scarce. Here we used manual deterministic tractography to investigate, for the first time, the integrity of the core white matter tracts defining the language connectivity network in non-verbal ASD (nvASD): the three segments of the arcuate (AF), the inferior fronto-occipital (IFOF), the inferior longitudinal (ILF) and the uncinate (UF) fasciculi, and the frontal aslant tract (FAT). A multiple case series of nine individuals with nvASD were compared to matched individuals with verbal ASD (vASD) and typical development (TD). Bonferroni-corrected repeated measure ANOVAs were performed separately for each tract—Hemisphere (2:Left/Right) × Group (3:TD/vASD/nvASD). Main results revealed (i) a main effect of group consisting in a reduction in fractional anisotropy (FA) in the IFOF in nvASD relative to TD; (ii) a main effect of group revealing lower values of radial diffusivity (RD) in the long segment of the AF in nvASD compared to vASD group; and (iii) a reduced volume in the left hemisphere of the UF when compared to the right, in the vASD group only. These results do not replicate volumetric differences of the dorsal language route previously observed in nvASD, and instead point to a disruption of the ventral language pathway, in line with semantic deficits observed behaviourally in this group.

**Supplementary Information:**

The online version contains supplementary material available at 10.1007/s00429-022-02474-1.

## Introduction

Non- or minimally verbal individuals with autism (nvASD) belong to the low-functioning section of autism spectrum disorder (ASD). They are defined by a severe expressive language deficit, which limits their spoken language acquisition to a handful of single words, with no compensation on the part of sign or written language (Tager-Flusberg and Kasari 2013). Current insights from magnetic resonance imaging are minimal and largely limited to two studies using diffusion tensor imaging to assess white matter (WM) structural connectivity, mainly focused on the exploration of WM tracts linked to mapping auditory information to articulatory motor representations. One of these studies revealed a reversal of a neurotypical left–right asymmetry of the arcuate fasciculus (AF) in four out of five non-verbal children with ASD (Wan et al. 2012). Similarly, when assessing treatment-based change in speech production of 10 minimally verbal children with ASD, an improvement during therapy was related to the integrity of both the left AF and right frontal aslant tract (FAT) (Chenausky et al. 2017). Further in line with this evidence, at least a subset of nvASD children have been reported to show childhood apraxia of speech (Chenausky et al. 2018), a developmental motor speech impairment (ASLHA 2021).

Language deviance in children and adults with nvASD, however, is not confined to expressive language. Language comprehension also falls far below the one expected from their chronological age (CA), and some evidence suggests that expressive and receptive language levels correlate in nvASD (Pickles et al. 2014; Chenausky et al. 2019; Hartley et al. 2019; Slusna et al. 2021). By definition, furthermore, nvASD are not characterized merely by a speech production deficit, but more broadly by an expressive language deficit, which as such reaches beyond the vocal-auditory modality. In the present study, therefore, we aimed to provide the first characterization in nvASD of the fronto-temporal language network as a whole.

This language network distributes information along both dorsal and ventral processing streams (Friederici 2011; Price 2012; Skeide and Friederici 2016). Broadly, the dorsal pathway is argued to support sound-to-motor mapping, that is, the mapping of auditory speech sounds to articulatory representations, while the ventral pathway sub-serves sound-to-meaning mapping, i.e., extracting meaning from auditory speech sounds (Hickok and Poeppel 2004). Structurally, the dorsal stream incorporates the superior longitudinal fasciculus (SLF)—AF complex, often referred to as SLF/AF, which can be segregated into one direct and two indirect segments (Catani et al. 2005). The SLF/AF underpins sensorimotor processes during speech production and perception (Hickok and Poeppel 2007; Rauschecker and Scott 2009) and is also argued to support higher-level syntactic processes (Friederici 2015). In addition, the FAT contributes to the dorsal stream with a function argued to be specific to speech production (Catani et al. 2013) or speech-specific cognitive control processes (Dick et al. 2014). Within the ventral processing stream, the inferior fronto-occipital fasciculus (IFOF) is regarded as a crucial pathway sub-serving semantic processes (Duffau et al. 2005; Saura et al. 2008). Running laterally to the IFOF, the inferior longitudinal fasciculus (ILF) has also been hypothesized to aid semantic processing, namely lexical retrieval (Herbet et al. 2019; Shin et al. 2019). Finally, the uncinate fasciculus (UF) potentially hosts local phrase structure building (Friederici et al. 2006) and might be recruited as an indirect pathway for semantics-related processes (Duffau et al. 2009; Harvey et al. 2013).

In the present study, we used manual deterministic tractography to reconstruct the entire aforementioned structural language connectome, comprising the IFOF, UF, FAT, ILF, and the three segments of the AF (long segment, anterior segment, posterior segment), in a case series of 9 nvASD children and adolescents. While this approach is highly labor-intensive and difficult to pursue in large sample sizes, smaller samples provide an opportunity to allow for an individualized approach to the neuroanatomy of each participant (López-Barroso et al. 2013), and the combination of dissection proposals from different authors for the selected tracts (Catani and Thiebaut de Schotten 2008; Fekonja et al. 2019). After reconstructing these pathways, we estimated their WM macro- and micro-structural characteristics by extracting their corresponding tract volume, fractional anisotropy (FA) and radial diffusivity (RD) measures bilaterally. Volume is a white matter macrostructure measure thought to reflect intrinsic characteristics like fiber-packing, myelin sheath state or tract-surrounding vasculature and glial architecture (Vaquero et al. 2021). In terms of microstructure, several diffusion measures can be extracted from DW-MRI. FA is probably the most used one [compared to other less sensitive measures such as MD (Winston 2012)] and, like volume, it has been showed to be very sensitive to individual differences (Vaquero et al. 2016). FA reflects the degree of anisotropy as it denotes the ratio of the variance of the eigenvalues to their mean (Winston 2012) and can be modulated by several factors such as axon geometry (axon diameter and axonal count), fiber organization and coherence, myelination, or membrane permeability (Winston 2012; Zatorre et al. 2012; Jones et al. 2013; Friedrich et al. 2020). However, summary parameters may not represent the full picture as changes along various directions can remain uncovered and it might not allow to determine the direction of change in case of reduction or increase in anisotropy (Aung et al. 2013). Therefore, we also extracted radial diffusivity (RD), which has received a growing interest in recent years (Ripollés et al. 2017; Elmer et al. 2019) and can provide better structural details of the state of the axons and myelin (Aung et al. 2013). RD has been related to several biological factors, such as number of axons and axon density and, specially, to the myelination degree (with a demyelination related to increased RD values) (Song et al. 2005; Zatorre et al. 2012; Ripollés et al. 2017). Since myelination as reflected in RD values can serve as indicator of the efficiency in the action potentials’ conduction along WM pathways, RD could be seen as an index of proper brain/cognitive processing for the tracts studied (Fields 2008; Ripollés et al. 2017).

To obtain benchmarks of the tracts’ macro- and microstructural measures, we also explored 9 typically developing (TD) and 9 verbal children with ASD (vASD), obtained from an online ASD neuroimaging database (ABIDE II), pair-matched on sex, age and handedness with our locally recruited group of nvASD. We hypothesized structural alterations in both ASD groups, showing deviance in the neural organization of language within both the dorsal and ventral streams. This was based on widespread structural anomalies along both of these routes previously documented in vASD cohorts (Travers et al. 2012; Li et al. 2019). In particular, there have been reports of a loss of hemispheric lateralization of the AF (Fletcher et al. 2010; Joseph et al. 2014; Liu et al. 2019), and of aberrant WM integrity in the UF associated with socio-affective deficits (Samson et al. 2016; Li et al. 2019), while some studies have also pointed to structural alterations in the IFOF and ILF (Jou et al. 2011; Aoki et al. 2013). By comparing nvASD to both a neuro-typical and a vASD group, we expected that a pattern continuous with that of vASD, but potentially more extended, might emerge in the more severe nvASD, though a differential pattern specific to nvASD could also transpire.

## Methods and materials

### Ethics approval

This study was approved by the corresponding institutional review board (CEIC Fundació Sant Joan de Déu; PIC-99-17). Written informed consent was obtained from legal guardians of all participants.

### Participants

Nine non- or minimally verbal school-aged children and adolescents diagnosed with ASD (nvASD, 3 females, mean age = 12.5 ± 3.23) were recruited from special schools in Barcelona, Spain. Recruitment criteria included: (a) a parent/center-reported ASD diagnosis confirmed during recruitment via the Autism Diagnostic Observation Schedule (ADOS) (Lord et al. 2012) and the Autism Diagnostic Interview-Revised (ADI-R) (Rutter et al. 2003); (b) an absence of phrase-level functional speech. To compare this sample with benchmarks across TD and verbal ASD (vASD), a database collected at the San Diego State University (SDSU) was used. Specifically, we included two control groups consisting of (i) nine typically developing (TD) children, and (ii) nine vASD children matched on age, sex and handedness. Recruitment criteria for vASD consisted of a clinical diagnosis of ASD confirmed by the ADIR-R, ADOS, and a DSM-5-based clinical judgment, while TD participants required a parent-reported absence of personal/family history of ASD or other neurological or psychiatric conditions. See Table 1 for demographic and neuropsychological data from all three groups.

**Table 1:** Demographic and neuropsychological participant profile

	TD Mean ± SD (*n* = 9)	vASD Mean ± SD (*n* = 9)	nvASD Mean ± SD (*n* = 9)	*p* value
Demographic information				
Sex (male/female)	6/3	6/3	6/3	1.000
Handedness (right/left)	8/1	8/1	8/1	1.000
Age at MRI acquisition (years; months)	12;6 ± 3.49	12;8 ± 3.07	12;6 ± 3.23	0.992
Neuropsychological profile				
Verbal mental age (VMA)/IQ	110.33 ± 10.33	93.56 ± 13.34	24.25 ± 15.74	–
Non-verbal IQ	108.44 ± 8.69	104.56 ± 16.38	63.75 ± 16.91	–
Diagnostic score (ADOS)	–	15.11 ± 2.76	17.88 ± 3.64	–

Demographic information—Data are means ± SD unless otherwise stated. Between-groups differences were explored using *χ*^2^ tests for sex and handedness and one-way ANOVA for age at MRI acquisition. Statistical tests confirmed the lack of significant different across the three groups. Neuropsychological profile—The neuropsychological tests applied were different for the three groups due to their intrinsic characteristics. Tests administered were: Verbal Mental Age (VMA)/IQ—Peabody Picture Vocabulary Test-III (PPVT-III) in nvASD, Wechsler Abbreviated Scale of Intelligence in vASD and TD; Non-Verbal IQ—Leiter International Performance Test-Revised (Leiter-R) in nvASD, Wechsler Abbreviated Scale of Intelligence in vASD and TD; ADOS—autism diagnostic observation schedule-2/-Adapted (ADOS-2/ADOS-A) in nvASD and vASD

Abbreviations: *TD* Typically development, *vASD* Verbal autism spectrum disorder, *nvASD* Non-verbal autism spectrum disorder, *IQ* intelligence quotient, *MA* mental age, *ADOS* autism diagnostic observation schedule

### MRI acquisition

Non-verbal ASD participants were scanned under anesthesia, as approved by the corresponding institutional review board (CEIC Fundació Sant Joan de Déu; PIC-99-17), on a Philips Ingenia 3 T scanner using a 64-channel head coil at the Sant Joan de Déu Hospital, Barcelona. Diffusion-weighted images (DWI) were acquired with a spin-echo echo-planar imaging (EPI) sequence (TR = 10,100 ms, TE = 102 ms, 64 axial slices, 36 directions, 90° flip angle, slice thickness = 2.1 mm, FOV = 23 cm, acquisition matrix = 112 × 112, voxel size = 2.05 mm^3^) with three non-diffusion (*b* = 0 s/mm^2^) and 36 diffusion weighted volumes (*b* = 1250 s/mm^2^). Data from TD and vASD subjects were collected on a GE 3 T Discovery MR750 scanner using an 8-channel head coil (UCSD–CFMRI). DWI were acquired with an EPI sequence (TR = 8500 ms, minimum TE by scanner protocol, 68 axial slices, 61 directions, slice thickness = 2.0 mm, FOV = 24 cm, acquisition matrix = 128 × 128, voxel size = 2.05 mm^3^) with one non-diffusion (b = 0 s/mm^2^) and 61 diffusion weighted volumes (*b* = 1250 s/mm^2^).

### MRI pre-processing

A visual inspection was performed by an expert for all data prior to the pre-processing to ensure the absence of any major artifact (due to acquisition errors, movement or others) that could not be corrected during the subsequent processing steps. All images were pre-processed using FMRIB Software Library (FSL www.fmrib.ox.ac.uk/fsl/fdt) and Diffusion Toolkit software (DTK) (Wang et al. 2007). DWI were processed as follows: (i) eddy-current correction using FMRIB’s Diffusion Toolbox (FDT), part of FMRIB Software Library (FSL www.fmrib.ox.ac.uk/fsl/fdt); (ii) brain extraction using FSL’s Brain Extractor Tool (Smith 2002; Smith et al. 2004; Woolrich et al. 2009) with 0.3 as threshold value; (iii) rotation of the b-vectors; (iv) reconstruction of the diffusion tensors using DTK (Wang et al. 2007); and (v) whole-brain deterministic tractography using DTK with 35° as maximum curvature and a minimum FA threshold of 0.2.

### Tract dissections

Manual deterministic tractography was performed focusing on the five main language-related tracts: arcuate (AF), inferior fronto-occipital (IFOF), inferior longitudinal (ILF), uncinate (UF) fasciculi, and frontal aslant tract (FAT). Tracts were dissected for each participant in native space, in both hemispheres, using Trackvis software (v.0.6.0.1, http://trackvis.org/) by manually placing Regions of Interest (ROI) as identified in previous reports (Catani and Thiebaut de Schotten 2008; Fekonja et al. 2019).

#### AF

The three segments of the AF were dissected using three ROIs drawn in a single slice as described in previous studies (Catani et al. 2005; Lopez-Barroso et al. 2013): a first ROI was delineated in the coronal view encompassing the fibers going to the inferior frontal gyrus (IFG) (including BA44 and 45); a second ROI was drawn in the axial plane covering the WM fibers traveling to the superior temporal gyrus; finally, a third ROI was depicted on the sagittal view, covering supra-marginal and angular gyri. These ROIs were combined to reconstruct the three subdivisions of the AF: the long (fronto-temporal), the anterior (fronto-parietal), and the posterior (temporo-parietal) segments.

#### FAT

To dissect the frontal aslant tract, two ROIs were delineated: the first was a spherical ROI of radius 8 mm located in the IFG and the second one was a single slice ROI placed in the WM of the superior frontal gyrus, encompassing fibers traveling to the Supplementary Motor Area (SMA) and pre-SMA (Catani et al. 2013).

#### ILF, UF & IFOF

For the delineation of the WM pathways supporting the ventral stream for language processing (i.e., ILF, IFOF and UF) (Hickok and Poeppel 2007; Rauschecker and Scott 2009), we used the combination of four ROIs according to previous publications (Catani and Thiebaut de Schotten 2008; Fekonja et al. 2019). The first ROI was placed axially at the level of the anterior temporal lobe (temporal ROI) spreading throughout an average of 5 slices; the second one on the anterior floor of the external/extreme capsule covering an average of 3 slices (frontal ROI); a third one on the region located between the occipital and temporal lobe (occipital ROI); and a fourth spherical ROI of radius 6.5 mm was placed in the middle temporal region, anterior to the radiation of the corpus callosum (temporo-occipital ROI). To define each of the tracts of interest, we applied a two-ROI approach: ILF was composed of fibers going through the temporal and occipital ROIs; streamlines going through both anterior and frontal ROIs were considered as part of the UF; finally, the fibers crossing the frontal and temporo-occipital ROIs formed the IFOF (following Fekonja’s method) (Fekonja et al. 2019).

Fekonja’s method of dissection was selected here for reconstructing the IFOF because we found it to be more permissive in the inclusion of fibers than other methods, generating a more plausible outcome for our type of data, processed following a diffusion tensor kind of analysis (as opposed to higher-resolution data that could be processed using spherical deconvolution methods, for instance). Nonetheless, and as stated in the main text, we additionally followed the Catani and Thiebaut de Schotten’s (2008) approach, which defines the IFOF as the fibers traveling through the frontal and occipital ROIs as described above. As expected, both dissection approaches generated similar results in our analyses. See the Online Resource 1 for details and comparison of the ANOVA test performed with the data extracted using each type of IFOF reconstruction.

Finally, artefactual fibers, if present in any of the tracts/hemispheres, were removed using exclusion ROIs, as is standard practice in manual reconstructions (Elmer et al. 2019; Vaquero et al. 2021).

To determine the microstructural measures to include in the main analyses, the whole brain’s fractional anisotropy (FA), radial diffusivity (RD), mean diffusivity (MD) and axial diffusivity (AD) values were extracted for each participant. Pearson’s correlations were then performed between all whole-brain microstructural measures (FA, MD, RD and AD) of all participants. Significant high correlations were found between all three directional microstructural measures: MD and RD (*r* = 0.972, *p* < 0.001), MD and AD (*r* = 0.974, *p* < 0.001), and AD and RD (*r* = 0.894, *p* < 0.001). By contrast, FA did not significantly correlate with any of the other three measures: MD (*r* = − 0.015, *p* = 0.939); AD (*r* = 0.193, *p* = 0.334); RD (*r* = − 0.228, *p* = 0.252). Based on these results, in previous language-related studies (Ripollés et al. 2017; Elmer et al. 2019), and to avoid redundancy, we focused only on one of the directional measures: RD, in addition to FA and volume, as our final measures of interest. As previously stated, dissections were performed in each participant’s native space. To control for potential variations in total brain volume, tract volume values were normalized by dividing each tract volume by the total WM volume (from the native space FA maps) for each participant. These normalized volume values were the ones included in the between-groups comparison analyses.

**Fig. 1:**
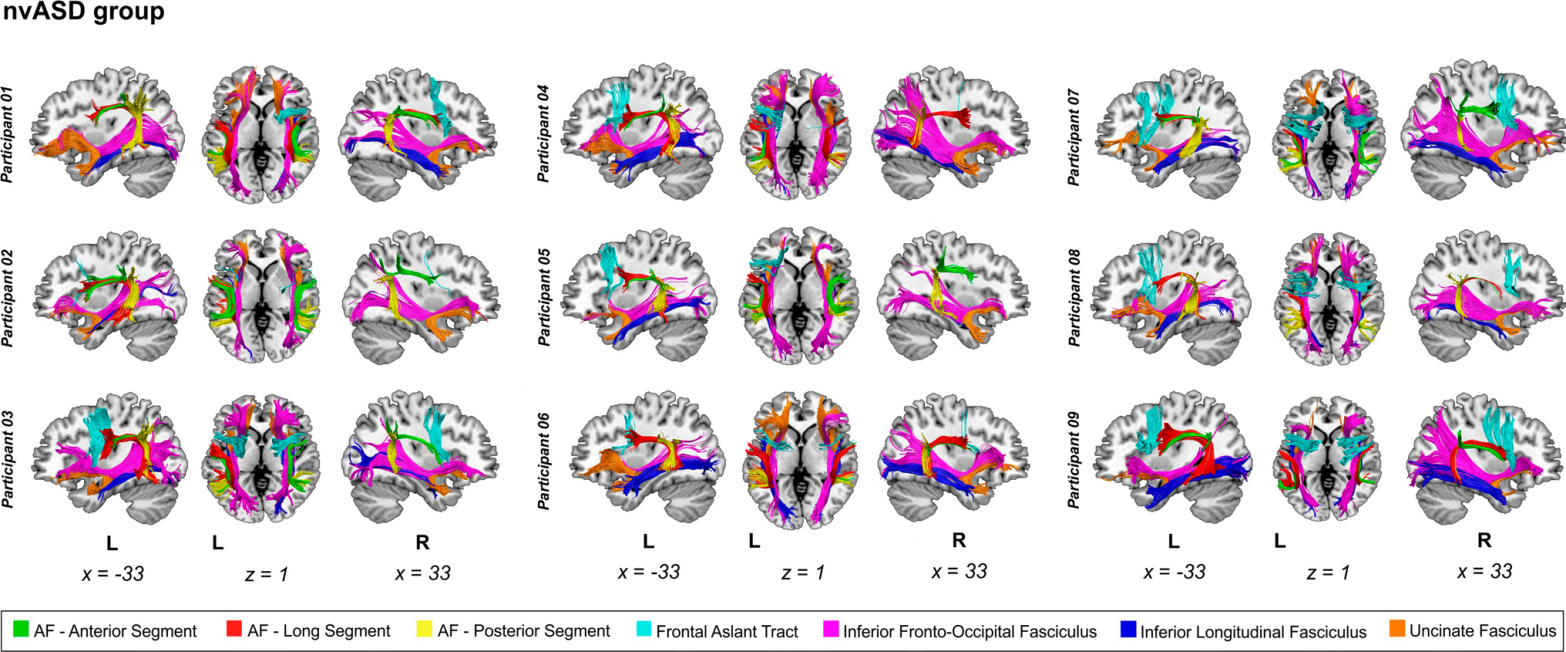
Dissections of nvASD participants. Manual deterministic tractography reconstructions from all participants of the nvASD group. Tracts reconstructed were the three segments of the arcuate fasciculus (AF) [Green = anterior, red = long, yellow = posterior segments], Frontal Aslant tract (FAT) [Cyan], Inferior Frontal Occipital Fasciculus (IFOF) [Purple], Inferior Longitudinal Fasciculus (ILF) [Dark blue] and Uncinate Fasciculus (UF) [Orange]. Abbreviations: *L* left. Montreal Neurological Institute space coordinates of the structural template slices are specified at the bottom of the image

**Fig. 2:**
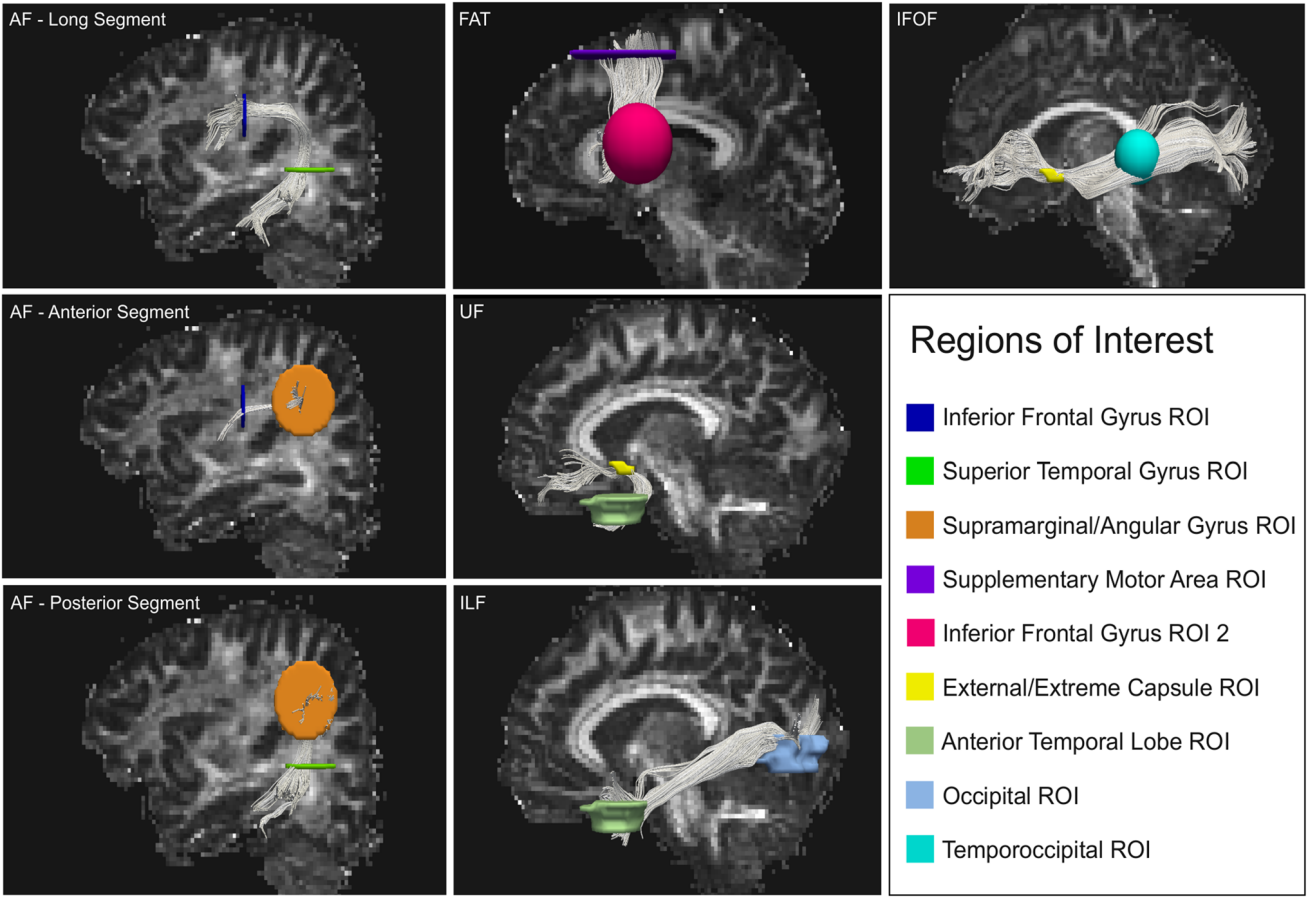
Regions of Interest placement examples. Regions of interest (ROI) placements for manual deterministic tractography reconstructions of the selected tracts. Tracts reconstructed were the three segments of the arcuate fasciculus (AF), Frontal Aslant tract (FAT), Uncinate Fasciculus (UF), Inferior Longitudinal Fasciculus (ILF) and Inferior Frontal Occipital Fasciculus (IFOF)

The dissections for all participants of the nvASD group are given in Fig. 1 and dissections of the vASD and TD participants can be found in the Online Resource 5. For visualization purposes, rendering of the streamlines was performed using the ‘tube’ render option of TrackVis with a radius of 0.15 mm. Examples of ROI placement are depicted in Fig. 2.

### Statistical analysis

Statistical analyses were performed using IBM SPSS software (v25.0). Hemisphere (2: Left/Right) x Group (3: TD/vASD/nvASD) repeated measures ANOVAs were performed separately for each tract (i.e., AF, FAT, IFOF, ILF, UF) and WM measure (volume, FA, RD), resulting in 15 ANOVAs (5 tracts per 3 measures). Bonferroni correction for multiple comparisons at *p* < 0.005 was applied and only results with a *p* value below this threshold will be presented below; for uncorrected trends see Online Resources 2, 3 and 4.

## Results

**Fig. 3:**
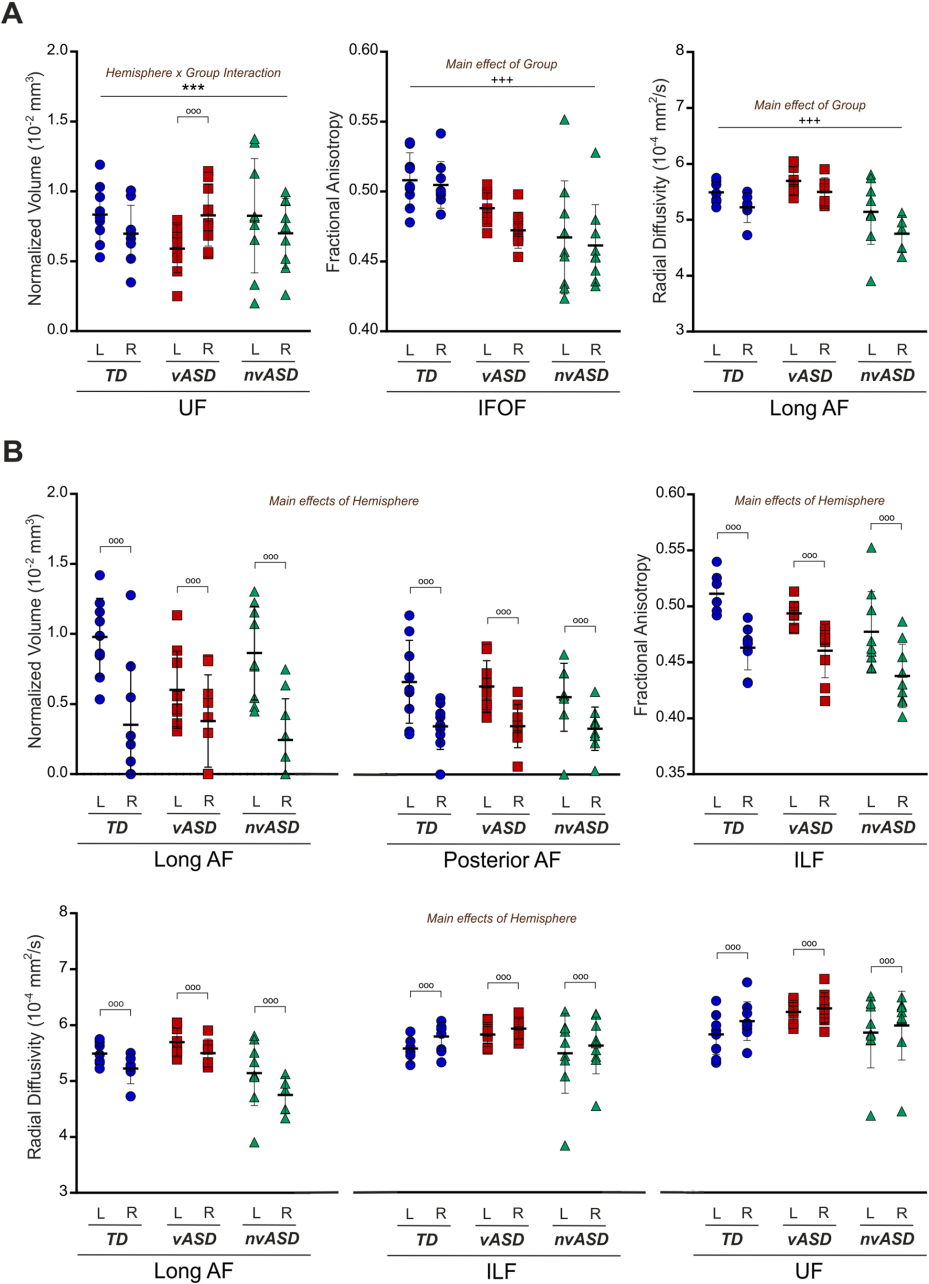
Structural connectivity results: normalized volume, fractional anisotropy and radial diffusivity. Significant results of the repeated measures ANOVA performed for the structural connectivity measures (normalized volume, FA and RD) extracted from each tract, with values for both hemispheres depicted in each group (blue circles correspond to TD participants, dark pink squares show vASD participants, and teal triangles represent nvASD participants). **A** Left graph shows the distribution of normalized volume values in the UF, marking the significant Hemisphere × Group interaction; central graph shows the distribution of FA values in the IFOF with the Main effect of Group specified; right graph depicts the distribution of RD values in the Long segment of the AF, with the Main effect of Group specified. **B** All the Main effects of Hemisphere found for all tracts and measures. Top row: Normalized volume values for the long segment of the AF (left) and posterior segment (center) of the AF, and FA values of the ILF (right). Bottom row: RD measures for the long segment of the AF (left), the ILF (center) and the UF (right). All results were Bonferroni-corrected (p < .005). Abbreviations: *UF* Uncinate fasciculus, *IFOF* Inferior frontal occipital fasciculus; *AF* Arcuate fasciculus, *ILF* Inferior longitudinal fasciculus, *L* Left, *R* Right

ANOVA results are detailed in the Online Resources 2, 3 and 4, and significant results and distributions are depicted in Fig. 3.

### Tract volume

A main effect of hemisphere was observed for the volume of the long [*F*(1,24) = 40.982, *p* < 0.001] and posterior (*F*(1,24) = 42.485, *p* < 0.001) segments of the AF, both showing larger volumes in the left compared to the right AF across all groups. Importantly, an interaction of hemisphere and group was observed in the UF (*F*(2,24) = 9.997, *p* < 0.001), showing a reduced volume in the left compared to the right UF, in the vASD group only, with Bonferroni-corrected *post hoc* tests confirming this effect (volume differences between left and right UF in vASD: *F*(1,24) = 12.486, *p* = 0.002; in TD: *F*(1,24) = 4.080, *p* = 0.055; in nvASD: *F*(1,24) = 3.469, *p* = 0.075).

### Fractional anisotropy

A main effect of hemisphere was found in the ILF [*F*(1,23) = 63.097, *p* < 0.001], where larger FA values were found in the left compared to the right hemisphere across the three groups. Moreover, a main effect of group was encountered in the IFOF [*F*(2,24) = 8.062, *p* = 0.002], showing a gradual tendency to decrease in FA in both ASD groups compared to TD individuals (TD > vASD > nvASD). Post hoc comparisons (Bonferroni-corrected) showed that this effect was driven by differences between TD and nvASD groups [*F*(2,24) = 8.062, *p* = 0.002], whereas the comparisons between TD and vASD [*F*(2,24) = 8.062, *p* = 0.061] or between vASD and nvASD [*F*(2,24) = 8.062, *p* = 0.448] groups did not reach significance.

### Radial diffusivity

A main effect of hemisphere was observed for the long segment of the AF [*F*(1,14) = 9.294, *p* = 0.009], driven by larger RD values in the left compared to the right AF. Relevantly, a main effect of group was also found in RD for the long segment of the AF [*F*(2,14) = 8.813, *p* = 0.003]. Bonferroni-corrected post hoc comparisons showed that this effect was driven by lower RD values in the nvASD group, resulting in significant differences between vASD and nvASD groups [*F*(2,14) = 9.294, *p* = 0.003], whereas the comparisons between TD and nvASD [*F*(2,14) = 9.294, *p* = 0.035] or between TD and vASD [*F*(2,14) = 9.294, *p* = 0.664] groups did not reach significance. On the other hand, the ILF [*F*(1,23) = 11.562, *p* = 0.002] and the UF [*F*(1,24) = 11.021, *p* = 0.003] also presented significant hemisphere effects but in this case showing larger RD values on the right compared to the left hemisphere.

## Discussion

This study aimed to investigate language-related WM structural connectivity alterations in nvASD individuals compared to matched verbal ASD (vASD) and typical development (TD) individuals. Manual DWI deterministic tractography was used for reconstruction of the main WM fiber tracks associated with language processing. We focused on individual volume, FA and RD measures as markers of white matter macro- and microstructural integrity of the tracts of interest and compared them between groups. The three main findings are, firstly, a main effect of group consisting in a reduction in FA in the IFOF in nvASD relative to the TD group; secondly, a main effect of group showing lower RD values in the long segment of the AF in nvASD compared to the vASD group; and finally, a significant interaction of hemisphere and group in the UF, which showed reduced volume in the left hemisphere when compared to the right only in the vASD group.

The reduction of FA in the IFOF in nvASD compared to TD individuals is a new finding. Although the exact involvement of the IFOF in language functions is still unclear, previous reports have demonstrated its role in reading, writing and attention (Catani and Thiebaut de Schotten 2008; Dorrichi et al. 2008), but it has first and foremost been considered as a crucial pathway sub-serving semantic processing (Catani and Thiebaut de Schotten 2008; Dick et al. 2014; Fekonja et al. 2019). In line with this, several lesion and tumor studies using electric stimulation have shown the relationship between IFOF integrity and proficiency in a semantic matching task (Sierpowska et al. 2019), a verbal fluency task (Almairac et al. 2015), and the number of semantic paraphasias (Duffau et al. 2005; Sierpowska et al. 2019), but not for semantic learning (Ripolles et al. 2017).

To understand the IFOF’s contribution in language processing, the anatomical course and terminations of the IFOF can be of great value. Recently, both DTI and anatomical post-mortem dissection studies have described the main course of the IFOF at the level of the insula and the temporal lobe (Catani and Thiebaut de Schotten, 2008; Martino et al. 2010), but more debate has been generated with respect to its anterior and posterior terminations. Sarubo et al. (2013) attempted to describe the frontal terminations of the IFOF by combining anatomical dissections and DWI. The authors proposed a division of the tract in two major components: a superficial one, terminating in the inferior frontal gyrus (IFG) and a deeper one, connecting with the middle frontal gyrus (MFG), dorso-lateral prefrontal cortex (DLPFC), the orbitofrontal cortex and the frontal pole. Similarly, Wu et al. (2016) used high-resolution diffusion tensor tractography to identify five subcomponents of the IFOF based on its frontal terminations [which overlapped greatly with those described by Sarubo et al. (2013)]. These results would support the idea of the IFOF as a ‘multi-function’ tract, with a clear involvement in language processing due to its role in conveying information to crucial language-related regions and nearby ones (IFG, MFG, DLPFC and orbitofrontal cortex). In most cases, these are associated to semantic processing functions (Plaza et al. 2008; Binder et al. 2009). Similarly, Martino et al. (2010) used post-mortem anatomical dissections to investigate and describe the posterior terminations of this tract. In this case, the authors also suggested the division of the IFOF into a superficial and a deeper component based on the posterior terminations. The former would project to the superior parietal lobe and posterior parts of the superior and middle occipital gyrus, whereas the latter would be associated with terminations in the inferior occipital gyrus and the posterior temporo-basal area. Again, the terminations of the IFOF in the associative extra-striate cortex and posterior temporo-basal area would further support the involvement of this tract in semantic functions (Price 2000; Vihla et al. 2006; Martino et al. 2010).

Despite this evidence, no study until now has attempted to elucidate the role of this pathway in a disorder with a clear semantic impairment such as individuals with nvASD. In standardized settings, language comprehension measures in this group have yielded scores far below those expected by individuals’ CA (Garrido et al. 2015; DiStefano et al. 2016; Chenausky et al. 2019; Slusna et al. 2021), and caregiver reports consistently document a lack of understanding or following of complex linguistic constructions (e.g., three-step instructions) in individuals with nvASD (Skwerer et al. 2016). Although children with nvASD show variation in how many single words they produce, there is evidence that those words are not semantically understood as carrying referential meaning (Preissler 2008), unlike what is seen already even in very young neuro-typical infants (Marno et al. 2015). In line with this, experimental assessments using EEG have uncovered anomalous patterns of lexico-semantic neural processing in a mixed group of nonverbal and preverbal children with ASD (Cantiani et al. 2016), effectively pointing to an aberrant rather than delayed language processing, in line with the neural patterns observed here. Although lexical semantic anomalies are seen throughout ASD (Tek et al. 2008; Arunachalam and Luyster 2016), these certainly do not reach the level of the essential absence of neuro-typical word use in nvASD, suggesting that ventral structural alterations of the IFOF may indeed be unique to nvASD.

In this study, we capitalized on manual dissection, despite it being labor-intensive and making larger samples difficult. This method was selected as it allowed a more suitable neuroanatomic approach for the research question of the study. First, manual dissections make the tract reconstruction adaptable to individual differences, which in the present case of developing brains (children and adolescents) is crucial, since most automatic dissection tools are based on adult anatomical landmarks/atlases. Second, we wanted to combine different authors’ proposals for dissecting the IFOF, a complex tract for which both anterior and posterior terminations are highly controversial. Despite the multiple possible frontal terminations discussed for this tract, all the streamlines are compacted when passing through the external/extreme capsule, so a first region of interest placed in this bottleneck should include all of the tract’s fibers, as suggested by Catani and Thiebaut de Schotten (2008). However, the posterior ROI proposed by these authors is a lot more restrictive as it does not encompass some of the parietal and superior occipital terminations observed post-mortem by other authors, such as Martino et al. (2010). Hence, we opted for a more inclusive ROI in the middle temporal gyrus, anterior to the radiation of the corpus callosum (Fekonja et al. 2019), comprising all the fibers coming from the temporal isthmus before they spread into their final cortical destination. The aim of this approach was to be as comprehensive as possible when selecting fibers, to ensure a complete and anatomically reliable characterization of the structural connectivity of this tract, which seems to be crucial for the understanding of this disorder. Nonetheless, very similar results were obtained when using the two ROIs proposed by Catani and Thiebaut de Schotten (2008) for the dissection of the IFOF as compared to the more comprehensive approach (see online resource 1).

Unlike in the case of our predictions for the ventral language pathway, our findings did not confirm our predictions based on previous literature in nvASD for structural alterations of the dorsal language pathway. These predictions were based on the study by Wan et al. (2012), who compared volume lateralization of the arcuate fasciculus between five completely non-verbal ASD and five TD children. Their results showed a rightward laterality (instead of the typical leftward asymmetry) in nvASD, which the authors argue could be critical for the language deficits observed in this group. However, our current results reveal lower values of RD in the long segment of the arcuate fasciculus in nvASD compared to vASD. RD can be defined as the magnitude of water diffusion perpendicular to the tract (Winklewski et al. 2018) and it has been suggested that a reduction in RD could translate into greater myelination and faster or more synchronized information transfer between brain regions (Ripollés et al. 2017). If so, this main effect of group found in the AF need not indicate an impairment in the dorsal language pathway for nvASD (as previously reported), but rather an enhanced information transfer efficiency.

The statistical and methodological limitations of our current data prevent us from a clear interpretation of this result. However, future studies could try to elucidate whether this enhanced microstructural organization found along the long segment in nvASD individuals is actually derived from an inherent between-group tract difference, or if it is due to a compensatory mechanism to overcome the problems derived from the alterations we have observed in the ventral pathway in this same group (i.e., reduced FA along the IFOF). Nevertheless, these results make evident the need of studying the language connectome as a whole—by means of different measures across several tracts—to try to understand group differences and better characterize vASD and nvASD structural connectivity patterns in a holistic way. In principle, several factors could explain the divergence between previous and our results concerning the nvASD group: a difference in the selection of the tractography method (probabilistic vs. deterministic), in the sample size (five vs nine participants per group), or even the inclusion criteria applied (completely vs. minimally verbal ASD children). In sum, while not ruling out dorsal route involvement, our results do not support that the severe language problems observed in nvASD can be solely due to problems of sensory-motor integration related to the AF and the dorsal processing route. Instead, they point to a greater deficit involving anomalous comprehension and semantic language processing.

Although it was not the original focus of this investigation, anomalies in the ventral language route were also found here for the vASD group. Specifically, higher volume of the UF on the right compared to the left hemisphere was observed in this group, a result that converges with previous findings in both children and adults with vASD (Catani et al. 2016; Samson et al. 2016; Li et al. 2019). Some of this previous work proposed that the maldevelopment of the UF, a tract connecting the lateral orbitofrontal cortex and Brodmann area 10 with the anterior temporal lobe (Von der Heide et al. 2013), is a potential neural substrate for the socio-affective deficits observed in this group (Samson et al. 2016; Li et al. 2019). Our vASD and nvASD individuals, however, shared a diagnosis and were selected so as to differ in language, not in socio-affective deficits. Further work is therefore required to corroborate what functions the UF supports. Given anomalies relating to the ventral route of language processing found in both ASD groups in our study, our results are consistent with a more localized ventral impact in vASD, as reflected by macrostructural alterations in a short and restricted associative bundle such as the UF, while nvASD shows a more global effect underpinned by a microstructural anomaly in the IFOF, a massive tract crossing the entire brain ventrally. Furthermore, as neural profiles between nvASD and vASD diverge, it is possible that nvASD should not be viewed as continuous with vASD, but as a relatively separate group within the autism spectrum, with distinct structural correlates.

There are a number of limitations to this study, which we were not able to supersede during the experiment. One main limitation is the acquisition of the neuroimaging data at two different scanning sites for TD and vASD groups on one hand, and nvASD group on another. This fact also implies different scanning protocols, and while two crucial neuroimaging parameters—like voxel size or *b* value—were matched, others, such as coil channels or TE/TR, were not. This fact may imply a bias due to the scanning protocol that cannot be dissociated from the main analyses and results. In fact, there appeared to be a slight global shift in FA and RD values for the nvASD group compared to the vASD and TD values (such as slightly lower values and higher standard deviations). These differences might be related to inherent microstructural differences in this group, and generalized scanner artifacts that resulted in this shift seem unlikely given the specificity of the patterns observed. Nevertheless, we cannot exclude the possibility that potential differences in the scanning conditions might have contributed to the observed differences. Also, the fact that dissections were performed in native space for every participant, extracting individual values from selected tracts, implies less methodological issues than voxel-based techniques performed at a group-level and implying potential registration errors. In line with this, it is important to note that very few previous neuroimaging studies have investigated nvASD participants due to the substantial difficulties of acquiring brain images of good quality in this population. If possible, future studies should try to overcome this methodological limitation by scanning at a single site or by obtaining brain data of a reduced number of subjects from both scanners (to compare and extract potential quantitative measures of control to add in the analyses). Another limitation is the reduced sample size, which, although larger than that of the key previous study (Wan et al. 2012), prevents definitive conclusions. Finally, as previously discussed, manual dissection was used for this study, but future work should try to expand the sample size and complement the analyses with other tractography methods like TRACULA (a global probabilistic approach—Yendiki et al. 2011), AFQ (an automated deterministic method—Yeatman et al. 2012), or tract-based spatial statistics (TBSS, to compare at group and voxel-based-like levels—Smith et al. 2006). This would help to better understand the neurobiological basis of this extreme side of the ASD spectrum, from which we know little in terms of structural neural underpinnings, despite its prevalence.

## Conclusion

Our investigation revealed a more complex pattern of WM structural differences in nvASD than the one expected from previous findings. Unlike the previously reported disruption of the dorsal language processing route, the key finding of the present study is a reduction of FA in the IFOF in nvASD compared to TD. These results suggest the disruption of the ventral language pathway as contributing to the severe language problems exhibited at this end of the autism spectrum, in line with behavioral findings of semantic deficits in this group. Although lower RD values were found for the long segment of the AF in the nvASD group relative to the vASD group, our results clearly suggest that further investigations should not merely be centered on the articulatory motor or dorsal route (only comprising tracts, such as the AF and FAT), but that a more comprehensive investigation of the language network is needed. We also observed an increased volume in the right compared to the left UF in vASD, possibly indicating a more localized ventral processing problem in this group, which, interestingly, did not generalize to nvASD.

## Supplementary Information

Below is the link to the electronic supplementary material.

Supplementary file1 (DOCX 34 KB)

Supplementary file2 (JPG 2088 KB)

## Data Availability

Anonymized data will be shared by request from any qualified investigator.

## References

[CR1] Almairac F, Herbet G, Moritz-Gasser S, de Champfleur N, Duffau H (2015). The left inferior fronto-occipital fasciculus subserves language semantics: a multilevel lesion study. Brain Struct Funct.

[CR2] Aoki Y, Abe O, Nippashi Y, Yamasue H (2013). Comparison of white matter integrity between autism spectrum disorder subjects and typically developing individuals: a meta-analysis of diffusion tensor imaging tractography studies. Mol Autism.

[CR3] Arunachalam S, Luyster RJ (2016). The integrity of lexical acquisition mechanisms in autism spectrum disorders: a research review. Autism Res.

[CR4] ASLHA: American Speech-Language-Hearing Association (2021) Childhood apraxia of speech. https://www.asha.org/public/speech/disorders/childhood-apraxia-of-speech/. Accessed 9 June 2021

[CR5] Aung W, Mar S, Benzinger T (2013). Diffusion tensor MRI as a biomarker in axonal and myelin damage. Imaging Med.

[CR6] Binder J, Desai R, Graves W, Conant L (2009). Where is the semantic system? A critical review and meta-analysis of 120 functional neuroimaging studies. Cereb Cortex.

[CR7] Cantiani C, Choudhury NA, Yu YH, Shafer VL, Schwartz RG, Benasich AA (2016). From sensory perception to lexical-semantic processing: an ERP study in non-verbal children with autism. PLoS ONE.

[CR8] Catani M, Thiebaut de Schotten M (2008). A diffusion tensor imaging tractography atlas for virtual in vivo dissections. Cortex.

[CR9] Catani M, Jones DK, Ffytche DH (2005). Perisylvian language networks of the human brain. Ann Neurol.

[CR10] Catani M, Mesulam MM, Jakobsen E (2013). A novel frontal pathway underlies verbal fluency in primary progressive aphasia. Brain.

[CR11] Catani M, Dell’Acqua F, Budisavljevic S (2016). Frontal networks in adults with autism spectrum disorder. Brain.

[CR12] Chenausky K, Kernbach J, Norton A, Schlaug G (2017). White matter integrity and treatment-based change in speech performance in minimally verbal children with autism spectrum disorder. Front Hum Neurosci.

[CR14] Chenausky KV, Tager-Flusberg H, Schlaug G (2018) Childhood apraxia of speech in minimally verbal children with ASD. Poster presented at the meeting of International Society for Autism Research annual meeting, Rotterdam 10.1002/aur.2006 PMC6203638 30230700

[CR13] Chenausky K, Brignell A, Morgan A, Tager-Flusberg H (2019). Motor speech impairment predicts expressive language in minimally verbal, but not low verbal, individuals with autism spectrum disorder. Autism Dev Lang Impair.

[CR15] Dick AS, Bernal B, Tremblay P (2014). The language connectome: new pathways, new concepts. Neuroscientist.

[CR16] DiStefano C, Shih W, Kaiser A, Landa R, Kasari C (2016). Communication growth in minimally verbal children with ASD: the importance of interaction. Autism Res.

[CR17] Dorrichi F, Thiebaut de Schotten M, Tomaiuolo F, Bartolomeo P (2008). White matter (dis)connections and gray matter (dys)functions in visual neglect: gaining insights into the brain networks of spatial awareness. Cortex.

[CR18] Duffau H, Gatignol P, Mandonnet E, Peruzzi P, Tzourio-Mazoyer N, Capelle L (2005). New insights into the anatomo-functional connectivity of the semantic system: a study using cortico-subcortical electrostimulations. Brain.

[CR19] Duffau H, Gatignol P, Moritz-Gasser S, Mandonnet E (2009). Is the left uncinate fasciculus essential for language? : a cerebral stimulation study. J Neurol.

[CR20] Elmer S, Hänggi J, Vaquero L, Olivé G, François C, Rodríguez-Fornells A (2019). Tracking the microstructural properties of the main white matter pathways underlying speech processing in simultaneous interpreters. Neuroimage.

[CR21] Fekonja L, Wang Z, Bährend I (2019). Manual for clinical language tractography. Acta Neurochir.

[CR22] Fields RD (2008). White matter in learning, cognition and psychiatric disorders. Trends Neurosci.

[CR23] Fletcher PT, Whitaker RT, Tao R (2010). Microstructural connectivity of the arcuate fasciculus in adolescents with high-functioning autism. Neuroimage.

[CR24] Friederici AD (2011). The brain basis of language processing: from structure to function. Physiol Rev.

[CR25] Friederici AD (2015). White-matter pathways for speech and language processing. Handbook of clinical neurology.

[CR26] Friederici AD, Bahlmann J, Heim S, Schubotz RI, Anwander A (2006). The brain differentiates human and non-human grammars: functional localization and structural connectivity. Proc Natl Acad Sci USA.

[CR27] Friedrich P, Fraenz C, Schlüter C, Ocklenburg S, Mädler B, Güntürkün O, Genç E (2020). The relationship between axon density, myelination, and fractional anisotropy in the human corpus callosum. Cereb Cortex.

[CR28] Garrido D, Carballo G, Franco V, García-Retamero R (2015). Language comprehension disorders in non-verbal children with autism spectrum disorders and their implications in the family quality of life. Rev Neurol.

[CR29] Hartley C, Trainer A, Allen ML (2019). Investigating the relationship between language and picture understanding in children with autism spectrum disorder. Autism.

[CR30] Harvey DY, Wei T, Ellmore TM, Hamilton AC, Schnur TT (2013). Neuropsychological evidence for the functional role of the uncinate fasciculus in semantic control. Neuropsychologia.

[CR31] Herbet G, Moritz-Gasser S, Lemaitre AL, Almairac F, Duffau H (2019). Functional compensation of the left inferior longitudinal fasciculus for picture naming. Cogn Neuropsychol.

[CR32] Hickok G, Poeppel D (2004). Dorsal and ventral streams: a framework for understanding aspects of the functional anatomy of language. Cognition.

[CR33] Hickok G, Poeppel D (2007). The cortical organization of speech understanding. Nature.

[CR150] Jones D, Knösche T, Turner R (2013). White matter integrity, fiber count, and other fallacies: the do's and don'ts of diffusion MRI. Neuroimage.

[CR34] Joseph RM, Fricker Z, Fenoglio A, Lindgren KA, Knaus TA, Tager-Flusberg H (2014). Structural asymmetries of language-related gray and white matter and their relationship to language function in young children with ASD. Brain Imaging Behav.

[CR35] Jou RJ, Mateljevic N, Kaiser MD, Sugrue DR, Volkmar FR, Pelphrey KA (2011). Structural neural phenotype of autism: Preliminary evidence from a diffusion tensor imaging study using tract-based spatial statistics. Am J Neuroradiol.

[CR36] Li Y, Zhou Z, Chang C (2019). Anomalies in uncinate fasciculus development and social defects in preschoolers with autism spectrum disorder. BMC Psychiatry.

[CR37] Liu J, Tsang T, Jackson L (2019). Altered lateralization of dorsal language tracts in 6-week-old infants at risk for autism. Dev Sci.

[CR38] López-Barroso D, Catani M, Ripollés P, Dell’Acqua F, Rodríguez-Fornells A, De Diego-Balaguer R (2013). Word learning is mediated by the left arcuate fasciculus. Proc Natl Acad Sci USA.

[CR39] Lord C, Rutter M, DiLavore P, Risi S, Gotham K, Bishop S (2012) Autism Diagnostic Observation Schedule, Second Edition (ADOS-2) Manual (Part I): Modules 1–4. 2nd Editio. Torrance, CA.: Western Psychological Services. https://psycnet.apa.org/doiLanding?doi=10.1037%2Ft17256-000. Accessed 10 June 2021

[CR40] Marno H, Farroni T, Vidal Dos Santos Y, Ekramnia M, Nespor M, Mehler J (2015). Can you see what I am talking about? Human speech triggers referential expectation in four-month-old infants. Sci Rep.

[CR41] Martino J, Brogna C, Robles SG, Vergani F, Duffau H (2010). Anatomic dissection of the inferior fronto-occipital fasciculus revisited in the lights of brain stimulation data. Cortex.

[CR42] Pickles A, Anderson DK, Lord C (2014). Heterogeneity and plasticity in the development of language: a 17-year follow-up of children referred early for possible autism. J Child Psychol Psychiatry Allied Discip.

[CR43] Plaza M, Gatignol P, Cohen H, Berger B, Duffau H (2008). A discrete area within the left dorsolateral prefrontal cortex involved in visual-verbal incongruence judgment. Cereb Cortex.

[CR44] Preissler MA (2008). Associative learning of pictures and words by low-functioning children with autism. Autism.

[CR45] Price CJ (2000). The anatomy of language: contributions from functional neuroimaging. J Anat.

[CR46] Price CJ (2012). A review and synthesis of the first 20 years of PET and fMRI studies of heard speech, spoken language and reading. Neuroimage.

[CR47] Rauschecker JP, Scott SK (2009). Maps and streams in the auditory cortex: nonhuman primates illuminate human speech processing. Nat Neurosci.

[CR48] Ripollés P, Biel D, Peñaloza C, Kaufmann J, Marco-Pallarés J, Noesselt T, Rodríguez-Fornells A (2017). Strength of temporal white matter pathways predicts semantic learning. J Neurosci.

[CR49] Rutter M, Le Couteur A, Lord C (2003) Autism diagnostic interview-revised. Western Psychological Services, Los Angeles, CA, 29(2003), 30

[CR50] Samson AC, Dougherty RF, Lee IA, Phillips JM, Gross JJ, Hardan AY (2016). White matter structure in the uncinate fasciculus: implications for socio-affective deficits in autism spectrum disorder. Psychiatry Res Neuroimaging.

[CR51] Sarubbo S, De Benedictis A, Maldonado IL, Basso G, Duffau H (2013). Frontal terminations for the inferior fronto-occipital fascicle: anatomical dissection, DTI study and functional considerations on a multi-component bundle. Brain Struct Funct.

[CR52] Saura D, Kreher BW, Schnell S (2008). Ventral and dorsal pathways for language. Proc Natl Acad Sci USA.

[CR53] Shin J, Rowley J, Chowdhury R (2019). Inferior longitudinal fasciculus’ role in visual processing and language comprehension: a combined MEG-DTI study. Front Neurosci.

[CR54] Sierpowska J, Gabarrós A, Fernández-Coello A, Camins À, Castañer S, Juncadella M, François C, Rodríguez-Fornells A (2019). White-matter pathways and semantic processing: intrasurgical and lesion-symptom mapping evidence. NeuroImage: Clin.

[CR55] Skeide MA, Friederici AD (2016). The ontogeny of the cortical language network. Nat Rev Neurosci.

[CR56] Skwerer DP, Jordan SE, Brukilacchio BH, Tager-Flusberg H (2016). Comparing methods for assessing receptive language skills in minimally verbal children and adolescents with autism spectrum disorders. Autism.

[CR57] Slusna D, Rodriguez A, Salvado B, Vicente A, Hinzen W (2021). Relations between language, non-verbal cognition and conceptualization in non- or minimally verbal individuals with ASD across the lifespan. J Autism Dev Disord.

[CR58] Smith SM (2002). Fast robust automated brain extraction. Hum Brain Mapp.

[CR59] Smith SM, Jenkinson M, Woolrich MW (2004). Advances in functional and structural MR image analysis and implementation as FSL. Neuroimage.

[CR60] Smith SM, Jenkinson M, Johansen-Berg H, Rueckert D, Nichols TE, Mackay CE, Watkins KE, Ciccarelli O, Cader MZ, Matthews PM, Behrens TE (2006). Tract-based spatial statistics: voxelwise analysis of multi-subject diffusion data. Neuroimage.

[CR61] Song SK, Yoshino J, Le TQ, Lin SJ, Sun SW, Cross AH, Armstrong RC (2005). Demyelination increases radial diffusivity in corpus callosum of mouse brain. Neuroimage.

[CR62] Tager-Flusberg H, Kasari C (2013). Minimally verbal school-aged children with autism spectrum disorder: the neglected end of the spectrum. Autism Res.

[CR63] Tek S, Jaffery G, Fein D, Naigles LR (2008). Do children with autism spectrum disorders show a shape bias in word learning?. Autism Res.

[CR64] Travers BG, Adluru N, Ennis C (2012). Diffusion tensor imaging in autism spectrum disorder: a review. Autism Res.

[CR65] Vaquero L, Rodríguez-Fornells A, Reiterer S (2016). The left, the better: white-matter brain integrity predicts foreign language imitation ability. Cereb Cortex.

[CR66] Vaquero L, Ramos-Escobar N, Cucurell D, François C, Putkinen V, Segura E (2021). Arcuate fasciculus architecture is associated with individual differences in pre-attentive detection of unpredicted music changes. Neuroimage.

[CR67] Vihla M, Laine M, Salmelin R (2006). Cortical dynamics of visual/ semantic vs. phonological analysis in picture confrontation. Neuroimage.

[CR68] Von Der Heide RJ, Skipper LM, Klobusicky E, Olson IR (2013). Dissecting the uncinate fasciculus: disorders, controversies and a hypothesis. Brain.

[CR69] Wan CY, Marchina S, Norton A, Schlaug G (2012). Atypical hemispheric asymmetry in the arcuate fasciculus of completely nonverbal children with autism. Ann N Y Acad Sci.

[CR70] Wang R, Benner T, Sorensen AG, Wedeen VJ (2007). Diffusion toolkit: a software package for diffusion imaging data processing and tractography. Proc Intl Soc Mag Reson Med.

[CR71] Winklewski P, Sabisz A, Naumczyk P, Jodzio K, Szurowska E, Szarmach A (2018). Understanding the physiopathology behind axial and radial diffusivity changes—what do we know?. Front Neurol.

[CR72] Winston GP (2012). The physical and biological basis of quantitative parameters derived from diffusion MRI. Quant Imaging Med Surg.

[CR73] Woolrich MW, Jbabdi S, Patenaude B (2009). Bayesian analysis of neuroimaging data in FSL. Neuroimage.

[CR74] Wu Y, Sun D, Wang Y, Wang Y (2016). Subcomponents and connectivity of the inferior fronto-occipital fasciculus revealed by diffusion spectrum imaging fiber tracking. Front Neuroanat.

[CR75] Yeatman JD, Dougherty RF, Myall NJ, Wandell BA, Feldman HM (2012). Tract profiles of white matter properties: automating fiber-tract quantification. PLoS ONE.

[CR76] Yendiki A, Panneck P, Srinivasan P, Stevens A, Zöllei L, Augustinack J, Wang R, Salat D, Ehrlich S, Behrens T, Jbabdi S, Gollub R, Fischl B (2011). Automated probabilistic reconstruction of white-matter pathways in health and disease using an atlas of the underlying anatomy. Front Neuroinform.

[CR77] Zatorre RJ, Fields RD, Johansen-Berg H (2012). Plasticity in gray and white: neuroimaging changes in brain structure during learning. Nat Neurosci.

