# Supplementary material for: Structural connectivity in ventral language pathways characterizes non-verbal autism
Source: Brain Struct Funct. 2022 Mar 14;227(5):1817–29. doi: 10.1007/s00429-022-02474-1 (PMC9098538; doi:10.1007/s00429-022-02474-1)
Supplement: Supplementary file 1 — Supplementary file1 (DOCX 34 KB) [file 429_2022_2474_MOESM1_ESM.docx]

**SUPPLEMENTARY DATA**

**Online Resource 1 – Comparison of output values (native tract volume & FA) for the two dissection methods applied to the IFOF reconstruction and ANOVA results.**

| **Measurement**  **& Tract** | **TD (n=9)** | **vASD (n=9)** | **nvASD (n=9)** | **ANOVA effect** | **F values** | **p (<.05)** |
| --- | --- | --- | --- | --- | --- | --- |
|  |  |  |  |  |  |  |
| Volume Left IFOF  Catani | 7.82 ± 2.38 | 7.56 ± 2.41 | 5.73 ± 2.64 | **H** | 5.316 | 0.030 |
|  |  |  |  | **G** | 0.933 | 0.407 |
| Volume Right IFOF  Catani | 8.77 ± 1.70 | 7.59 ± 2.78 | 8.13 ± 2.80 | **H * G** | 1.988 | 0.159 |
| Volume Left IFOF  Fekonja | 11.62 ± 3.47 | 13.18 ± 4.16 | 10.36 ± 4.51 | **H** | 3.927 | 0.059 |
|  |  |  |  | **G** | 0.059 | 0.943 |
| Volume Right IFOF  Fekonja | 14.04 ± 3.70 | 11.87 ± 3.34 | 14.29 ± 3.68 | **H * G** | 3.357 | 0.052 |
| FA Left IFOF  Catani | 0.513 ± 0.016 | 0.492 ± 0.013 | 0.477 ± 0.041 | **H** | 0.727 | 0.402 |
|  |  |  |  | **G** | 6.354 | 0.006 |
| FA Right IFOF  Catani | 0.511 ± 0.018 | 0.488 ± 0.011 | 0.472 ± 0.028 | **H * G** | 0.050 | 0.951 |
| FA Left IFOF  Fekonja | 0.508 ± 0.020 | 0.488 ± 0.011 | 0.467 ± 0.040 | **H** | 6.925 | 0.015 |
|  |  |  |  | **G** | **8.062** | **0.002*** |
| FA Right IFOF  Fekonja | 0.505 ± 0.017 | 0.472 ± 0.013 | 0.462 ± 0.041 | **H * G** | 1.461 | 0.252 |

**Online Resource 1**- Hemisphere (2: Left/Right) x Group (3: TD / vASD / nvASD) repeated measures ANOVAs were performed separately for each dissection method and for the volume and FA measures in the IFOF. Mean ± SD are shown for each type of dissection (Catani et al., 2008 vs. Fekonja et al., 2019) in each hemisphere (rows) organized by group (columns). Significant p-values are marked with the * sign. Abbreviations: FA = Fractional Anisotropy; TD = Typical Development; vASD = Verbal Autistic Spectrum Disorder; nvASD = Non-Verbal Autistic Spectrum Disorder; AF = Arcuate Fasciculus; FAT = Frontal Aslant Tract; IFOF=Inferior Fronto-Occipital Fasciculus; ILF = Inferior Longitudinal Fasciculus; UF = Uncinate Fasciculus; H = Hemisphere main effect; G = Group main effect; H*G = Hemisphere by Group Interaction.

**Online Resource** **2 - Details of ANOVA analyses for the Relative Volume measures**

| **Measurement**  **& Tract** | **TD (n=9)** | **vASD (n=9)** | **nvASD (n=9)** | **ANOVA effect** | **F values** | **p (<0.005)** |
| --- | --- | --- | --- | --- | --- | --- |
|  |  |  |  |  |  |  |
| Rel. Vol. Left Anterior Segment AF | 2.5e-3 ± 2.4e-3 | 2.63e-3 ± 2.3e-3 | 2.07e-3 ± 2e-3 | **H** | 3.710 | 0.066 |
|  |  |  |  | **G** | 0.442 | 0.648 |
| Rel. Vol. Right Anterior Segment AF | 4.1e-3 ± 2.4e-3 | 2.88e-3 ± 2.3e-3 | 2.84e-3 ± 2e-3 | **H * G** | 0.716 | 0.499 |
| Rel. Vol. Left Long Segment AF | 9.78e-3 ± 2.8e-3 | 6.02e-3 ± 2.8e-3 | 8.64e-3 ± 3.3e-3 | **H** | **40.982** | **<0.001*** |
|  |  |  |  | **G** | 1.021 | 0.375 |
| Rel. Vol. Right Long Segment AF | 3.52e-3 ± 4.4e-3 | 3.79e-3 ± 3.3e-3 | 2.45e-3 ± 2.9e-3 | **H * G** | 3.038 | 0.067 |
| Rel. Vol. Left Posterior Segment AF | 6.62e-3 ±  3e-3 | 6.29e-3 ± 1.8e-3 | 5.53e-3 ± 2.4e-3 | **H** | **42.485** | **<0.001*** |
|  |  |  |  | **G** | 0.320 | 0.729 |
| Rel. Vol. Right Posterior Segment AF | 3.44e-3 ± 1.7e-3 | 3.46e-3 ± 1.6e-3 | 3.27e-3 ± 1.6e-3 | **H * G** | 0.406 | 0.671 |
| Rel. Vol. Left FAT | 2.43e-3 ± 2.2e-3 | 5.07e-3 ± 2.3e-3 | 5.88e-3 ± 4.1e-3 | **H** | 0.662 | 0.424 |
|  |  |  |  | **G** | 1.040 | 0.369 |
| Rel. Vol. Right FAT | 3.99e-3 ± 2.4e-3 | 4.55e-3 ± 3.7e-3 | 3.49e-3 ± 2.7e-3 | **H * G** | 4.207 | 0.027 |
| Rel. Vol. Left IFOF | 1.82e-2 ± 5.3e-3 | 2e-2 ±  6e-3 | 1.57e-2 ± 6.7e-3 | **H** | 4.172 | 0.052 |
|  |  |  |  | **G** | 0.271 | 0.765 |
| Rel. Vol. Right IFOF | 2.24e-2 ± 6.8e-3 | 1.8e-2 ±  4.1e-3 | 2.19e-2 ± 6.3e-3 | **H * G** | 3.406 | 0.050 |
| Rel. Vol. Left ILF | 5.67e-3 ± 4.1e-3 | 7.72e-3 ± 3.8e-3 | 6.84e-3 ± 2.9e-3 | **H** | 0,313 | 0.581 |
|  |  |  |  | **G** | 0.503 | 0.611 |
| Rel. Vol. Right ILF | 6.33e-3 ± 2.7e-3 | 6.8e-3 ±  3.5e-3 | 5.8e-3 ± 3.4e-3 | **H * G** | 0.492 | 0.617 |
| Rel. Vol. Left UF | 8.34e-3 ± 2.1e-3 | 5.9e-3 ±  1.7e-3 | 8.26e-3 ± 4.1e-3 | **H** | 0.041 | 0.842 |
|  |  |  |  | **G** | 0.164 | 0.849 |
| Rel. Vol. Right UF | 6.97e-3 ±  2e-3 | 8.29e-3 ± 2.2e-3 | 7e-3 ±  2.5e-3 | **H * G** | **9.997** | **<0.001*** |

**Online Resource 2-** Hemisphere (2: Left/Right) x Group (3: TD / vASD / nvASD) repeated measures ANOVAs were performed separately for each tract for the relative volume (Rel. Vol.) measure. Means ± SD are shown for each tract and hemisphere (rows) organized by group (columns). Significant p-values are marked with the * sign. Abbreviations: TD = Typical Developing Children; vASD = Verbal Autistic Spectrum Disorder Children; nvASD = Non-Verbal Autistic Spectrum Disorder Children; AF = Arcuate Fasciculus; FAT = Frontal Aslant Tract; IFOF=Inferior Fronto-Occipital Fasciculus; ILF = Inferior Longitudinal Fasciculus; UF = Uncinate Fasciculus; H = Hemisphere main effect; G = Group main effect; H*G = Hemisphere by Group Interaction.

**Online Resource 3 – Details of ANOVA analyses for Fractional Anisotropy (FA) measures**

| **Measurement**  **& Tract** | **TD (n = 9)** | **vASD (n = 9)** | **nvASD (n = 9)** | **ANOVA effect** | **F values** | **p (<.05)** |
| --- | --- | --- | --- | --- | --- | --- |
|  |  |  |  |  |  |  |
| FA Left Anterior Segment AF | 0.454 ± 0.03 | 0.434 ± 0.03 | 0.396 ± 0.05 | **H** | 2.956 | 0.105 |
|  |  |  |  | **G** | 4.383 | 0.030 |
| FA Right Anterior Segment AF | 0.458 ± 0.02 | 0.448 ± 0.04 | 0.415 ± 0.04 | **H * G** | 0.353 | 0.708 |
| FA Left Long Segment AF | 0.483 ± 0.01 | 0.457 ± 0.03 | 0.461 ± 0.04 | **H** | 2.263 | 0.155 |
|  |  |  |  | **G** | 1.785 | 0.204 |
| FA Right Long Segment AF | 0.493 ± 0.04 | 0.471 ± 0.01 | 0.472 ± 0.03 | **H * G** | 0.039 | 0.962 |
| FA Left Posterior Segment AF | 0.459 ± 0.03 | 0.456 ± 0.02 | 0.442 ± 0.04 | **H** | 0.111 | 0.742 |
|  |  |  |  | **G** | 2.730 | 0.087 |
| FA Right Posterior Segment AF | 0.473 ± 0.02 | 0.441 ± 0.03 | 0.450 ± 0.02 | **H * G** | 1.837 | 0.183 |
| FA Left FAT | 0.430 ± 0.020 | 0.441 ± 0.021 | 0.426 ± 0.034 | **H** | 2.690 | 0.117 |
|  |  |  |  | **G** | 0.630 | 0.543 |
| FA Right FAT | 0.432 ± 0.031 | 0.423 ± 0.034 | 0.410 ± 0.043 | **H * G** | 0.991 | 0.389 |
| FA Left IFOF | 0.508 ± 0.020 | 0.488 ± 0.011 | 0.467 ± 0.040 | **H** | 6.925 | 0.015 |
|  |  |  |  | **G** | **8.062** | **0.002*** |
| FA Right IFOF | 0.505 ± 0.017 | 0.472 ± 0.013 | 0.462 ± 0.041 | **H * G** | 1.461 | 0.252 |
| FA Left ILF | 0.511 ± 0.018 | 0.494 ± 0.010 | 0.477 ± 0.036 | **H** | **63.097** | **<0.001*** |
|  |  |  |  | **G** | 4.373 | 0.025 |
| FA Right ILF | 0.461 ± 0.020 | 0.460 ± 0.024 | 0.438 ± 0.028 | **H * G** | 0.901 | 0.420 |
| FA Left UF | 0.454 ± 0.030 | 0.423 ± 0.020 | 0.418 ± 0.033 | **H** | 8.044 | 0.009 |
|  |  |  |  | **G** | 4.348 | 0.024 |
| FA Right UF | 0.433 ± 0.021 | 0.420 ± 0.020 | 0.406 ± 0.033 | **H * G** | 1.520 | 0.239 |

**Online Resource 3**- Hemisphere (2: Left/Right) x Group (3: TD / vASD / nvASD) repeated measures ANOVAs were performed separately for each tract for the fractional anisotropy measure. Mean ± SD are shown for each tract and hemisphere (rows) organized by group (columns). Significant p-values are marked with the * sign. Abbreviations: FA = Fractional Anisotropy; TD = Typical Developing Children; vASD = Verbal Autistic Spectrum Disorder Children; nvASD = Non-Verbal Autistic Spectrum Disorder Children; AF = Arcuate Fasciculus; FAT = Frontal Aslant Tract; IFOF=Inferior Fronto-Occipital Fasciculus; ILF = Inferior Longitudinal Fasciculus; UF = Uncinate Fasciculus; H = Hemisphere main effect; G = Group main effect; H*G = Hemisphere by Group Interaction.

**Online Resource 4 – Details of ANOVA analyses for Radial Diffusivity (RD) measures**

| **Measurement**  **& Tract** | **TD (n=9)** | **vASD (n=9)** | **nvASD (n=9)** | **ANOVA effect** | **F values** | **p (<0.005)** |
| --- | --- | --- | --- | --- | --- | --- |
|  |  |  |  |  |  |  |
| RD Left Anterior Segment AF | 5.56e-4 ± 2.1e-5 | 5.84e-4 ± 2.7e-5 | 5.67e-4 ± 4.1e-5 | **H** | 5.757 | 0.029 |
|  |  |  |  | **G** | 1.493 | 0.254 |
| RD Right Anterior Segment AF | 5.5e-4 ± 1.6e-5 | 5.78e-4 ± 3.1e-5 | 5.49e-4 ±  4.9­e-5 | **H * G** | 0.891 | 0.430 |
| RD Left Long  Segment AF | 5.5e-4 ± 2.2e-5 | 5.67e-4 ± 2.9e-5 | 4.9e-4 ±  5.7e-5 | **H** | **9.294** | **0.009*** |
|  |  |  |  | **G** | **8.813** | **0.003*** |
| RD Right Long Segment AF | 5.22e-4 ± 2.7e-5 | 5.5e-4 ±  2.5e-5 | 4.75e-4 ± 3.3e-5 | **H * G** | 0.347 | 0.712 |
| RD Left Posterior Segment AF | 5.59e-4 ± 2.2e-5 | 5.69e-4 ± 3.2e-5 | 5.38e-4 ± 6.7e-5 | **H** | 0.835 | 0.371 |
|  |  |  |  | **G** | 1.850 | 0.181 |
| RD Right Posterior Segment AF | 5.42e-4 ± 2.3e-5 | 5.78e-4 ± 3.5e-5 | 5.29e-4 ±  7.2­e-5 | **H * G** | 1.471 | 0.251 |
| RD Left FAT | 5.76e-4 ± 2.9e-5 | 5.81e-4 ± 2.8e-5 | 5.24e-4 ± 6.8e-5 | **H** | 1.954 | 0.178 |
|  |  |  |  | **G** | 5.185 | 0.015 |
| RD Right FAT | 5.79e-4 ± 3.8e-5 | 6.02e-4 ± 3.8e-5 | 5.22e-4 ± 6.1e-5 | **H * G** | 1.635 | 0.220 |
| RD Left IFOF | 5.49e-4 ± 2.2e-5 | 5.75e-4 ± 1.8e-5 | 5.5e-4 ±  7.5e-5 | **H** | 0.756 | 0.393 |
|  |  |  |  | **G** | 1.754 | 0.195 |
| RD Right IFOF | 5.5e-4 ± 2.4e-5 | 5.86e-4 ± 1.6e-5 | 5.5e-4 ±  5.5e-5 | **H * G** | 0.599 | 0.558 |
| RD Left ILF | 5.57e-4 ± 1.8e-5 | 5.82e-4 ± 2.2e-5 | 5.49e-4 ± 7.1e-5 | **H** | **11.562** | **0.002*** |
|  |  |  |  | **G** | 1.588 | 0.226 |
| RD Right ILF | 5.82e-4 ± 2.5e-5 | 5.93e-4 ± 1.8e-5 | 5.63e-4 ± 5.1e-5 | **H * G** | 0.757 | 0.480 |
| RD Left UF | 5.83e-4 ± 3.6e-5 | 6.23e-4 ± 2.1e-5 | 5.86e-4 ± 6.3e-5 | **H** | **11.021** | **0.003*** |
|  |  |  |  | **G** | 1.817 | 0.184 |
| RD Right UF | 6.07e-4 ± 3.5e-5 | 6.3e-4 ±  2.8e-5 | 5.99e-4 ± 6.2e-5 | **H * G** | 1.358 | 0.276 |

**Online Resource 4**- Hemisphere (2: Left/Right) x Group (3: TD / vASD / nvASD) repeated measures ANOVAs were performed separately for each tract for the Radial Diffusivity measure. Mean ± SD are shown for each tract and hemisphere (rows) organized by group (columns). Significant p-values are marked with the * sign. Abbreviations: RD = Radial Diffusivity; TD = Typical Developing Children; vASD = Verbal Autistic Spectrum Disorder Children; nvASD = Non-Verbal Autistic Spectrum Disorder Children; AF = Arcuate Fasciculus; FAT = Frontal Aslant Tract; IFOF=Inferior Fronto-Occipital Fasciculus; ILF = Inferior Longitudinal Fasciculus; UF = Uncinate Fasciculus; H = Hemisphere main effect; G = Group main effect; H*G = Hemisphere by Group Interaction.

**Online Resource 5 – Dissections of vASD and TD participants**

**Online Resource 5** Manual deterministic tractography reconstructions from all participants of the TD (top) and vASD (bottom) groups. Tracts reconstructed were the three segments of the arcuate fasciculus (AF) [Green = anterior, red = long, yellow = posterior segments], Frontal Aslant tract (FAT) [Cyan], Inferior Frontal Occipital Fasciculus (IFOF) [Purple], Inferior Longitudinal Fasciculus (ILF) [Dark blue] and Uncinate Fasciculus (UF) [Orange]. Abbreviations: L, left. Montreal Neurological Institute space coordinates of the structural template slices are specified at the bottom of the image.
