# Supplementary figures and images for: Structural connectivity in ventral language pathways characterizes non-verbal autism
Source: Brain Struct Funct. 2022 Mar 14;227(5):1817–29. doi: 10.1007/s00429-022-02474-1 (PMC9098538; doi:10.1007/s00429-022-02474-1)

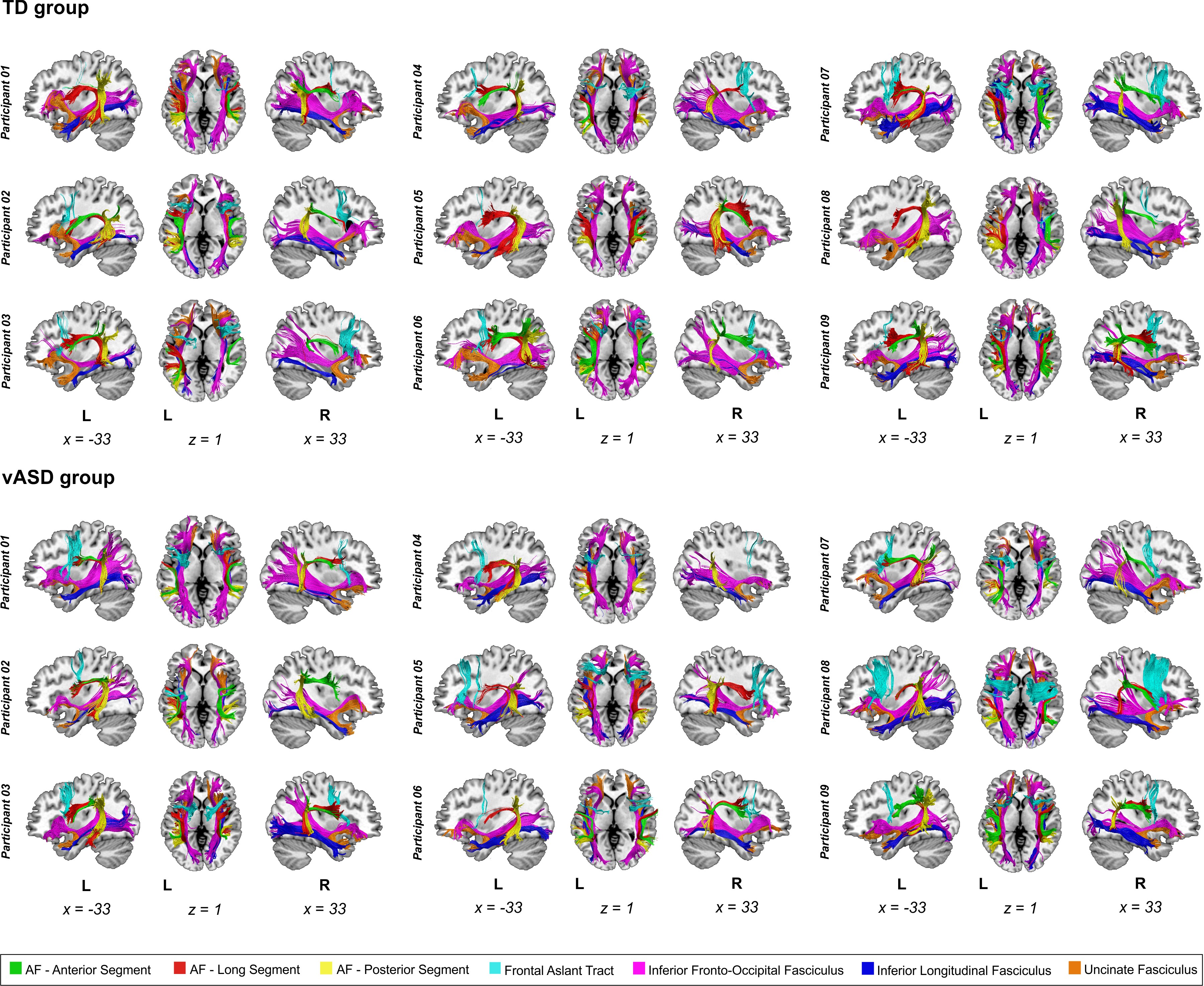

Supplement: Supplementary file 2 — Supplementary file2 (JPG 2088 KB) [file 429_2022_2474_MOESM2_ESM.jpg]
